# Mammalian and Avian Host Cell Influenza A Restriction Factors

**DOI:** 10.3390/v13030522

**Published:** 2021-03-22

**Authors:** Joe McKellar, Antoine Rebendenne, Mélanie Wencker, Olivier Moncorgé, Caroline Goujon

**Affiliations:** 1 Institut de Recherche en Infectiologie de Montpellier, CNRS, Université de Montpellier, CEDEX 5, 34293 Montpellier, France; joe.mckellar@irim.cnrs.fr (J.M.); antoine.rebendenne@irim.cnrs.fr (A.R.); 2 Centre International de Recherche en Infectiologie, INSERM/CNRS/UCBL1/ENS de Lyon, 69007 Lyon, France; melanie.wencker@inserm.fr

**Keywords:** influenza virus, restriction factors, interferon, innate immunity

## Abstract

The threat of a new influenza pandemic is real. With past pandemics claiming millions of lives, finding new ways to combat this virus is essential. Host cells have developed a multi-modular system to detect incoming pathogens, a phenomenon called sensing. The signaling cascade triggered by sensing subsequently induces protection for themselves and their surrounding neighbors, termed interferon (IFN) response. This response induces the upregulation of hundreds of interferon-stimulated genes (ISGs), including antiviral effectors, establishing an antiviral state. As well as the antiviral proteins induced through the IFN system, cells also possess a so-called intrinsic immunity, constituted of antiviral proteins that are constitutively expressed, creating a first barrier preceding the induction of the interferon system. All these combined antiviral effectors inhibit the virus at various stages of the viral lifecycle, using a wide array of mechanisms. Here, we provide a review of mammalian and avian influenza A restriction factors, detailing their mechanism of action and in vivo relevance, when known. Understanding their mode of action might help pave the way for the development of new influenza treatments, which are absolutely required if we want to be prepared to face a new pandemic.

## 1. Introduction

Influenza viruses are segmented, negative, single stranded RNA viruses that are members of the *Orthomyxoviridae* family. Influenza viruses are classified into four genera: A, B, C and D (IAV, IBV, ICV and IDV) [1]. In this review, we will be focusing solely on IAV. IAV finds its reservoir in waterfowl, where it is generally asymptomatic [2], but it also circulates in poultry and certain mammalian species, including humans, pigs and horses [3]. IAV are classified based on their haemagglutinin (HA) and neuraminidase (NA) surface proteins. To date, 18 HA and 11 NA subtypes have been discovered, if we include the influenza A-like bat viruses although they have been shown to unexpectedly differ from avian and human IAV [4,5]. IAV is a seasonal virus and the causative agent of the flu, a usually mild illness causing fever, coughs, muscle aches and fatigue, but can become severe through the development of complications, such as pneumonia, possibly leading to death. This virus is known to undergo two different evolution processes: antigenic drift and antigenic shift. The IAV RNA polymerase has a low fidelity, consistently introducing novel mutations into the viral genome. Antigenic drift is a slow process where mutations are introduced into the HA and NA segments and accumulate over time. As a consequence, more recent circulating strains differ from those circulating during past seasons and can evade antibodies produced during those seasons if changes in the viral antigens HA and NA are sufficient. Antigenic shift is a more major event, where two different viruses infect the same cell and hybrid progeny viruses are produced, possessing a mix of parental viral segments, also called reassortment [6]. This can allow for the emergence of new subtypes, to which the human population is immunologically naïve, potentially leading to IAV pandemics (mainly due to new and/or different HA segments) [7]. Hence, four naturally occurring pandemics have arisen since the beginning of the 20th century. The infamous 1918 “Spanish flu” (named as such for being first reported in Spanish news outlets due to the fact that media of countries involved in World War I were under censure) was caused by a H1N1 virus that infected over a third of the world’s population, killing over 50 million. Other pandemics include the 1957 H2N2 “Asian flu” killing over 2 million, the 1968 H3N2 “Hong Kong flu” killing over 1 million and the more recent 2009 H1N1 “Swine flu” killing around 200,000 (the latter was, thankfully, milder than seasonal flu). Genetic analysis suggests that the 1918 H1N1 virus originated from direct transfer of a whole avian strain into humans [8]. In contrast, the two following pandemics (H2N2 and H3N2) occurred by reassortment between the circulating human strain at the time (H1N1 and H2N2, respectively) and avian influenza viruses. As for the virus responsible for the 2009 pandemic (pH1N1), it was a complicated reassortant whose combination of genomic segments were unique, but all segments of the virus were present in viruses found in swine [9,10]. Also noteworthy, but different to the previous naturally occurring pandemics, was that of 1977, also called the “Russian flu”, where the culprit was a virus that was identical to the H1N1 virus that had circulated in humans in the 1950’s [11]. The most probable explanation for the unlikely occurrence of reappearance of a strain identical to the one circulating two decades prior is that of the accidental release of this virus from a frozen source. Hence, IAV pandemics emerge in a seemingly random manner. To date there are only a few approved antiviral treatments against flu, to which IAV can easily escape [12,13,14,15], as well as vaccines against seasonal strains (currently pH1N1, H3N2 as well as influenza B lineages). Vaccines are efficient when fully matching the circulating strains, however, they take time to produce and would not be ready until months within a new pandemic. Therefore, we are currently not ready to face a severe influenza pandemic and the current COVID-19 crisis is a cruel reminder of the damage that can be caused by respiratory viruses with pandemic potential.

This review has the goal of detailing the main intrinsic and innate defenses humans and other hosts, such as ducks and chickens, possess to defend themselves from IAV infection. These defenses come in the form of restriction factors, which are antiviral proteins expressed by the host cell that restrict or impair viruses at various stages of their replication cycle. These restriction factors can be constitutively expressed and/or induced as a result of viral sensing and consequent signaling. Understanding these natural defenses could potentially unlock ideas that might help pave the way for new treatments, necessary in the uncertainty of future IAV pandemics.

## 2. Influenza A Virus Life Cycle

**Figure 1:**
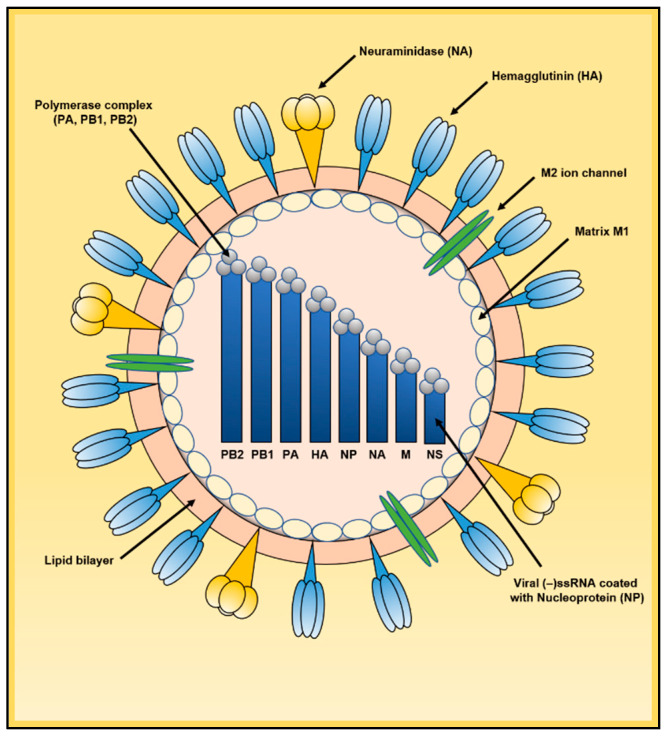
Annotated schematic representation of an IAV virion.

To understand the different restriction factors that inhibit IAV, a general understanding of the viral life cycle is necessary. When IAV (Figure 1) encounters a target cell, the HA surface glycoprotein interacts with a sialylated receptor on the cell plasma membrane [16,17]. Avian strains preferentially bind to α-2.3-linked sialic acids whereas human strains to α-2.6-linked sialic acids [18,19]. After attachment, the virus enters the cell through epsin1-dependent clathrin-mediated endocytosis, clathrin-independent endocytosis or through macropinocytosis [20,21,22]. Now inside the cell, the viral particle is localized in an endocytic vesicle. As this vesicle progresses through its lifecycle, acidification of the endosomal pH activates the surface M2 proton pump, acidifying the viral particle [23,24]. This decrease in pH fragilizes the matrix, allowing uncoating [25]. The acidification of the endosomal vesicle is also responsible for the multi-step fusogenic transformation of HA [26,27]. The HA fusion peptide then mediates the fusion of the cellular endosomal membrane together with the viral lipid envelope, allowing exit of the eight viral ribonucleoproteins (vRNPs) into the cytoplasm. vRNPs are constituted of a negative strand genomic RNA segment coated with viral nucleoprotein (NP) molecules and bound to the viral polymerase. Each vRNP acts as an autonomous transcription-replication unit once it reaches the cell nucleus. vRNPs enter the nucleus using the active importin α/β1 pathway mediated by the viral protein NP that possesses a Nuclear Localization Signal (NLS) that interacts with importin α [28,29].

Once the vRNPs are in the nucleus, viral messenger RNAs (mRNAs) are produced from the genomic viral RNA (vRNA) template by the resident viral RNA-dependent RNA polymerase (RdRp). This is a primed process, using primers from cellular mRNAs being transcribed by the cellular RNA polymerase II [30]. Polymerase Basic 2 (PB2), part of the tripartite viral polymerase along with Polymerase Acidic (PA) and Polymerase Basic 1 (PB1), binds the 5′ caps of cellular mRNAs. Following this, the PA endonuclease cleaves the 5′ end of the cellular mRNA, which releases a capped 9-17 nucleotide primer that is used to initiate the transcription of the viral mRNA using viral genomic RNA as a template [31]. This process is called cap-snatching. The viral mRNAs also possess polyA tails, just as cellular mRNAs, but that are produced by the viral polymerase. The newly produced mRNAs are exported into the cytoplasm and translated by cellular ribosomes, ending in the production of viral proteins. The viral polymerase subunits as well as NP, M1 and NS2/NEP proteins are then trafficked back to the nucleus. Occurring at the same time as mRNA synthesis, the viral polymerase is also responsible for vRNA replication. The viral polymerase transcribes the negative vRNA into positive complementary RNA (cRNA) in a non-primed process. The newly formed cRNA associates with novel NP, PB1, PB2 and PA molecules into complementary RNPs (cRNPs). Following this, the cRNPs undergo replication to create new negative vRNA copies which associate into novel vRNPs. For further details on the transcription and replication steps, see [32,33,34].

The newly formed vRNPs undergo export into the cytoplasm in a CRM1-dependent manner, mediated by a daisy-chain of vRNP-M1-NS2-CRM1 [35,36,37]. Once in the cytoplasm, vRNPs are next trafficked towards the plasma membrane in a Rab11-dependent process that relies on endoplasmic reticulum remodeling [38,39] and undergo assembly [40]. Ongoing questions in the lifecycle of IAV are how vRNPs are transferred to the plasma membrane, and how they organize themselves inside the viral particle into the classical “7+1” formation [41]. This highly coordinated process is followed by viral budding and egress. Release of the newly formed viral particle is catalyzed by NA that cleaves the sialic acids retaining the particle on the cell membrane.

## 3. Sensing and Interferon Response

### 3.1. Sensing and Interferon Response in Mammalian Species

**Figure 2:**
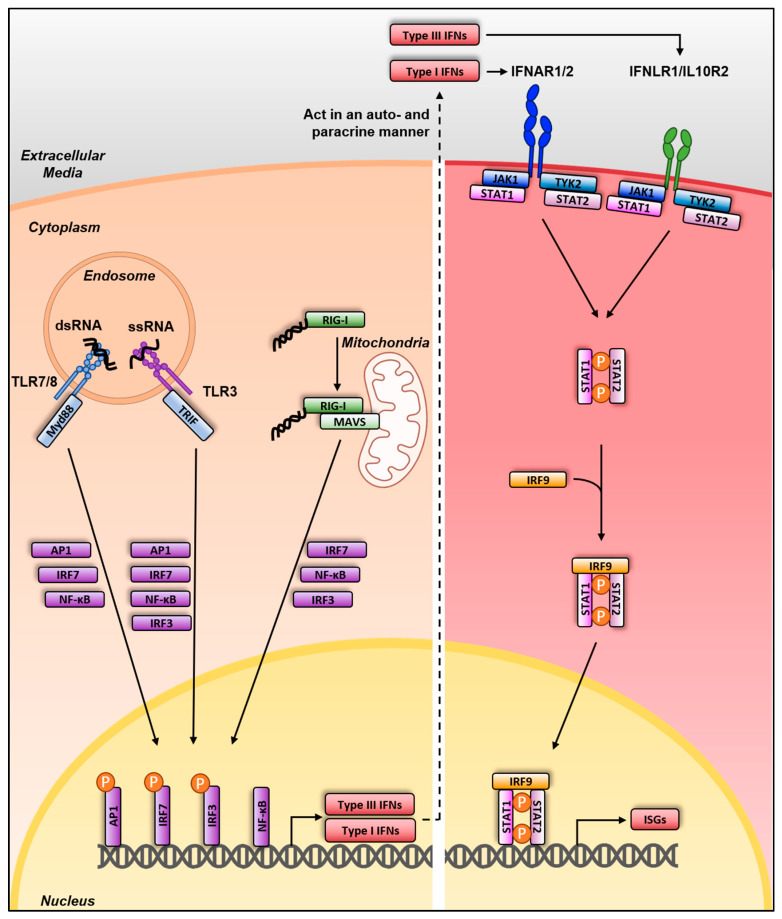
Schematic representation of IAV sensing and subsequent IFN signaling.

The detection of viral infections by the innate immune system occurs through the recognition of pathogen-associated molecular patterns (PAMPs) by pattern recognition receptors (PRRs). PAMPs are either present in invading pathogens or are generated during infection. Viral PAMPs usually correspond to specific nucleic acid structures of the viral genome not found in host cells or viral replication intermediates such as double-stranded RNA (dsRNA). Distinct classes of PRRs are important for influenza virus recognition and induced inflammation (Figure 2): the Toll-Like receptors (TLR), the Retinoic acid-inducible gene I (RIG-I) receptor, the NOD-like receptor family member NOD-, LRR- and pyrin-containing 3 (NLRP3) inflammasome and the Z-DNA binding protein 1 (ZBP1) (recently reviewed in [42]). These PRRs share different subcellular localizations and act at different stages of the viral replication cycle. Indeed, NLRP3, RIG-I and ZBP1 are located in the cytoplasm, whereas TLR3, 7 and 8 are present within endosomes [42].

Activation of these PRRs triggers signaling cascades leading to the production and secretion of type I and III interferons (IFNs), pro-inflammatory cytokines, eicosanoids and chemokines, which can act in a paracrine and autocrine manner. In particular, type I and III IFNs are potent antiviral mediators leading to the expression of hundreds of IFN-stimulated genes (ISGs), which induce an antiviral state [43]. Type I and III IFNs are notably expressed after PAMP recognition by RIG-I and TLR3/7/8, shortly described hereafter.

RIG-I is expressed by all cell types and activated upon binding to short dsRNA and RNA harboring di- or tri-phosphorylated 5′ ends. Following RNA binding to RIG-I, the latter is ubiquitinylated by TRIM25 or RIPLET E3 ubiquitin ligases, which are required for RIG-I activation and allow its higher-order oligomerization [44]. Then, RIG-I interacts with mitochondrial antiviral-signaling protein (MAVS), promotes its oligomerization and the formation of a signaling hub called signalosome required for downstream signaling. RIG-I is considered as the main immune sensor for IAV and recognizes its genomic RNA [45]. RIG-I importance was confirmed in in vivo studies in a mouse model. Indeed, type I IFN production was impaired in *Rig-I*^−/−^ mice infected by IAV, which correlated with delayed viral clearance [46]. Of note, mice are non-natural hosts for influenza, however, they are frequently used as models to study influenza-mediated pathogenesis given the large variety of genetic tools available.

TLR3 recognizes dsRNA and is expressed in various cell types including dendritic cells (DCs) and lung epithelial cells, the latter being target host cells of IAV. Numerous studies have shown the important role of TLR3 in IAV sensing. Indeed, in human bronchial epithelial cells, IAV infection leads to the activation of TLR3 and subsequent production of proinflammatory cytokines [47]. However, infection of *Tlr3*^−/−^ mice with IAV resulted in higher viral production in lungs but also a diminished proinflammatory cytokine response, which is actually less detrimental for IAV infected mice [48]. These results suggest that defense mechanism against IAV requires a tight regulation of immune responses in order to control IAV replication without exacerbated inflammatory responses. In that context, TLR3, while promoting viral clearance, could also participate in IAV-mediated pathogenesis.

TLR7 is mainly expressed by plasmacytoid DCs (pDCs) and lung epithelial cells whereas TLR8 is expressed by immune cells such as monocytes, macrophages and DCs. Classically, binding of single-stranded RNA by TLR7/8 in endosomes leads to their activation and the induction of their downstream signaling pathway in a Myeloid differentiation primary response 88 (MyD88) adaptor-dependent manner (Figure 2). Mice deficient for *Myd88* are more susceptible to IAV infection when sublethal doses where used for wild type animals [49]. However, the involvement of TLR7/8 in IAV immune sensing remains debated. Indeed, another study using *Myd88*^−/−^ mice showed no requirement of MyD88 in protection against IAV infection using lethal doses of IAV even for the wild-type mice, which could explain the discrepancy between the two studies [48]. Moreover, pDCs, which are the professional IFN producers, were surprisingly shown to not impact the course of IAV infection in mice [50].

Following engagement of RIG-I and TLR3/7/8, several transcription factors such as IRF3, IRF7 (in pDCs or in cells pre-exposed to IFN), AP1 and NF-κB are activated and translocate to the nucleus, leading to the transcription of type I/III *IFN* genes. Type I and III IFNs are then secreted and act in a paracrine and autocrine manner. The type I IFN receptor (composed of IFNAR1 and IFNAR2 subunits) is ubiquitously expressed, whereas the type III IFN receptor (composed of IFNLR1 and IL10R2 subunits) is preferentially expressed on mucosal epithelial cells, liver cells and some myeloid cells [51]. Upon binding to their cognate receptors, type I and III IFNs activate a Janus kinase (JAK)/signal transducer and activator of transcription (STAT) pathway. This signaling cascade results in the transcription of several hundreds of ISGs, some having antiviral and immunomodulatory properties, and in the establishment of an antiviral state in infected and neighboring cells.

Type I and III IFNs are well known to potently inhibit IAV replication in humans and in mice, both in vitro and in vivo through the expression of ISGs (reviewed in [52]). Moreover, IAV infection has also been shown to be restricted by type I IFNs in vitro in bat cells [53]. In line with this, mice models where IFN signaling is abrogated or exogenous IFNs are administrated, demonstrated the important role of both type I [54,55,56,57,58] and type III [59], [60] IFNs in vivo in IAV clearance and in the control of influenza-induced pathogenesis. Type I and III IFNs induce a similar subset of ISGs, with type I IFN leading to a more rapid induction and decline of ISG expression [43,61]. Importantly, type III IFNs are produced earlier than type I IFNs following IAV infection of mice, which leads to viral inhibition without inducing inflammation [61,62].

Of note, the importance of type I and III IFNs antiviral activities was also evidenced in humans. Indeed, exogenous prophylactic administration of IFNα seemed to reduce the severity of influenza infection [63]. Moreover, in humans, carried inborn errors in genes involved in the type I/III IFN pathway such as *TLR3* [64], *IRF7* [65] or *IRF9* [66] were reported in some life-threatening influenza patients. This highlights a critical role of type I and III IFNs in influenza disease severity and in the control of influenza replication.

Furthermore, the role of type I and III IFNs extends beyond a role in limiting viral replication. Indeed, type I and III IFNs are important to shape adaptative immune responses. Mice lacking type III IFN receptors show defective DC responses, ultimately preventing the optimal generation of effector CD8 T-cells [67,68], protective memory T-cell responses against IAV [67] and optimal antibody responses [68]. This effect of type III IFN appears indirect and depends, at least in part, on the secretion of thymic stromal lymphopoietin (TSLP) by microfold cells (M cells) in the upper airways [68], which promotes migration of resident CD103^+^ DCs to draining lymph nodes and germinal center reactions. Thus, type III IFNs via TSLP potentiate the establishment of adaptative immune responses [68] (reviewed in [61]).

Similarly, type I IFN favors memory CD8 T-cell cytolytic activity in an antigen-independent manner, by promoting the expression of granzyme B [69]. Furthermore, mice that lack STAT1, which fail to signal through all three types of IFN receptors, do not succeed to generate an IgG2a response and present a significant bias toward T_H_2 (T helper 2) differentiation and IgE response, which is at the origin of an exacerbated lung pathology [70].

### 3.2. Sensing and Interferon Response in Avian Species

The Galliformes order, which includes chickens, are characterized by the absence of RIG-I [71], which has been proposed to be at the origin of the high susceptibility of chickens to RNA viruses compared to ducks [72]. Despite the fact that RIG-I is the main sensor for IAV in mammalians, an IFN response is mounted in chickens infected by highly pathogenic influenza virus strains, potentially due to sensing of viral RNAs by RIG-I-like receptors MDA-5 and LGP2 [73,74,75]. Moreover, in chickens, only TLR3 and 7 are involved in the recognition of RNA viruses given that TLR8 is pseudogenized [76]. Type I and III IFNs are nevertheless produced in response to the activation of these PRRs, which inhibit IAV replication in vitro, in ovo and in vivo [77,78,79].

### 3.3. Viral Antagonism of Innate Immune Sensing by IAV

IAV has evolved various proteins such as NS1, PB1-F2 or PA-X to counteract the induction of innate immune responses either by inhibiting innate immune sensing or by inhibiting signaling downstream of type I or III IFN receptors (reviewed in [42,80]).

NS1 inhibits RIG-I-mediated immune sensing through several mechanisms. For instance, NS1, by interacting with dsRNA through its RNA binding domain, sequesters RIG-I ligands [81,82,83]. Furthermore, NS1 has been shown to interact with several proteins such as the E3 ubiquitin ligases TRIM25 or RIPLET, preventing RIG-I activation [84,85,86].

PB1-F2 is encoded from a +1 open reading frame (ORF) of the PB1 gene as a result of a leaky ribosomal scanning in some IAV strains. PB1-F2 plays pleiotropic effects, such as the enhancement of viral polymerase activity [87], the induction of cell death [88] or the modulation of innate immune signaling [89]. Concerning the latter, PB1-F2 has been shown to interact with the transmembrane domain of MAVS, leading to the dissipation of the mitochondrial membrane potential, which is essential for MAVS-mediated signaling. Moreover, association of PB1-F2 with MAVS could also impair its oligomerization at mitochondria, which is required for downstream signaling [90,91].

PA-X is encoded by IAV segment 3 via +1 ribosomal frameshifting, generated by ribosomal pausing on a rare CGU codon. PA-X shares the first 191 amino acids with PA, containing the endonuclease domain [92,93]. PA-X expression induces a general inhibition of host cell translation. This host cell shut-off is due to the degradation of host PolII-generated transcripts by the endonucleolytic domain in coordination with the 5′ to 3′-exonuclease Xrn1, leading to, among other effects, the inhibition of innate immune response [94,95,96,97].

## 4. IAV Restriction Factors

**Figure 3:**
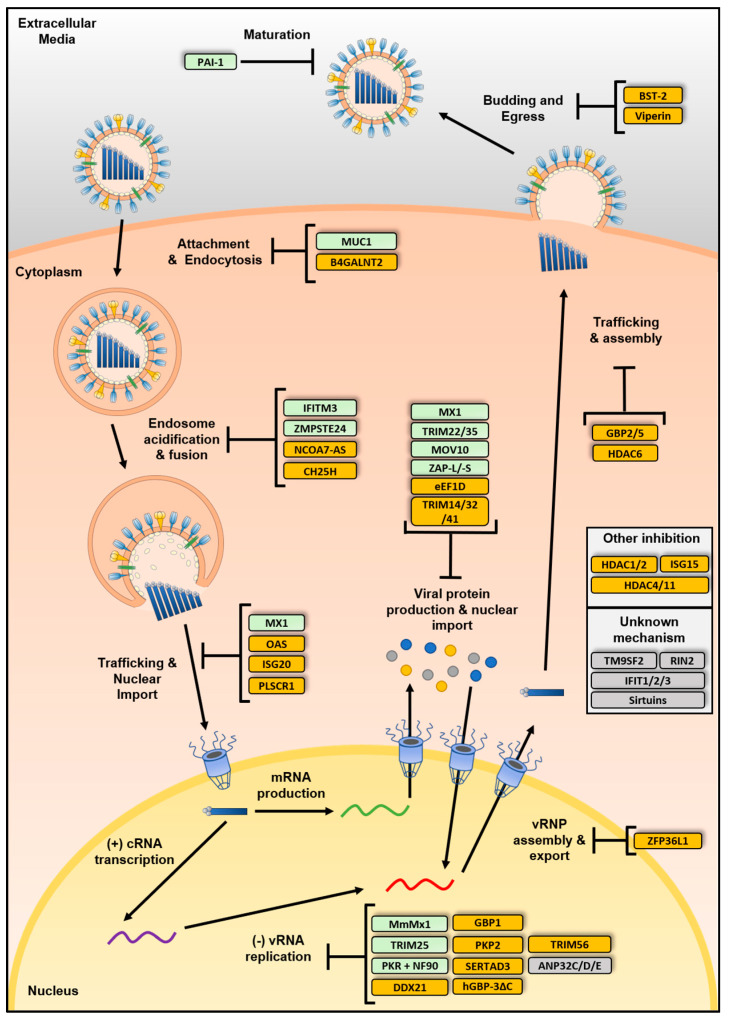
Schematic representation of IAV life cycle with the host cell restriction factors that target each step. Factors highlighted in green are those that are generally accepted or that have consequent data backing them, in orange factors that need further investigation to confirm their role as IAV restriction factors for instance in vivo (e.g., NCOA7-AS) and in grey, those for which very little data is available.

Restriction factors come in many forms but with the common effect of inhibiting viral replication. These proteins can act at various stages of the viral lifecycle and have also started emerging as being able to potentiate viral sensing and regulate antiviral signaling pathways. In this part of the review, we will focus on restriction factors that are able to inhibit IAV at various stages of its life cycle, be they constitutively expressed or induced following viral sensing. Figure 3 gives an overview of all restriction factors described here and the step they inhibit.

### 4.1. Restriction Factors Inhibiting Viral Entry

Viral entry into a host cell is an absolutely critical step in the life cycle of viruses, only possible if the adequate receptors are present at the cell surface. Interestingly, a number of restriction factors act at this step and are reviewed hereafter.

#### 4.1.1. IFITMs: Major, Pan-Viral Restriction Factors Preventing Cytosolic Entry

Interferon-induced transmembrane proteins (IFITMs) are small transmembrane proteins [98,99] that are evolutionary conserved across vertebrates [100,101]. In humans, the IFITM family is composed of five genes (*IFITM1*; *IFITM2*; *IFITM3*; *IFITM5*; *IFITM10*), whereas in mice, two additional genes (*Ifitm6*; *Ifitm7*) have been described. Generally, vertebrate IFITM families can be divided into immunity-related IFITM (IR-IFITM), IFITM5 and IFITM10. Initially, IFITM3 was identified in a siRNA screen to identify host factors modulating IAV infection [102]. IFITM1/2/3 proteins have since been shown to exert an antiviral activity against a wide range of viruses (reviewed in [103]). IFITM proteins are thought to be type IV transmembrane proteins based on epitope mapping in cells [104,105,106] and preliminary structural studies [107]. IFITMs are mostly located in early and late endosomes, and lysosomes for IFITM2/3, as shown by immunofluorescence studies [108,109] and live cell imaging [110,111]. However, IFITM1 lacks the AP2 sorting motif (YXXΦ) at the N-terminus and is rather located at the plasma membrane. Both the antiviral mechanism and specificity of IFITMs are linked to their cellular localization, with IFITM1 being more active against viruses that enter the target cell at the plasma membrane, whereas IFITM2/3 preferentially inhibit viruses entering target cells through endocytosis. Of note, IFITM3 can be incorporated into nascent viral particles and prevent viral spread by inhibiting subsequent viral fusion with a new target cell, as shown in the case of HIV-1 [112,113].

IFITM proteins are known to prevent cytosolic entry of the targeted viruses and a number of hypotheses to explain their activity, mostly based on the study of IFITM3, have emerged (reviewed in [99]). Overexpression of IFITM3 leads to an expansion of late endocytic compartments (Rab7- and LAMP1-positive compartments) along with their over-acidification, which suggests that IFITM3 could have a role in controlling pH-dependent viral entry [114]. It has also been proposed that IFITM3 inhibits hemifusion and lipid mixing [115]. However, it was later shown that IFITM3 prevents IAV cytosolic entry without inhibiting hemifusion, by affecting the formation of the fusion pore [116]. Interestingly, the impact on the formation of the fusion pore was later confirmed and IFITM3-positive vesicles were shown to fuse with vesicles containing incoming virions before hemifusion. This phenomenon was specific to viruses restricted by IFITM3 only and leads to an increased rate of virus trafficking to late endocytic compartments [111,117].

IFITM proteins possess intramembrane (IMD) and transmembrane (TMD) domains separated by an intracellular loop (ICL) and variable N- and C-terminal domains. All these domains play an important role in anti-IAV activity (as reviewed in [103]). Indeed, the CD225 domain, which is highly conserved among them, comprises the IMD, the TMD and the ICL domains and is essential for antiviral activity [118]. Moreover, deletion of the N-terminal domain of IFITM3 resulted in an impaired ability to inhibit IAV [118]. Specific motifs have been shown to be important for the antiviral activity, namely the ^20^YXXΦ^23^ motif in IFITM2/3, which is important for correct subcellular localization [119] or the ^81^SVKS^84^ motif of IFITM3 in the CIL domain, which is essential for IAV inhibition [120]. IFITM proteins are highly modified by post-transcriptional modifications (PTMs), which modulate their antiviral activity. Indeed, murine and human IFITM3 have been reported to be S-palmitoylated on cysteines 71, 72 and 105, and this is important for antiviral activity [104,121]. S-palmitoylation is a reversible PTM, which increases IFITM3 hydrophobicity and affinity for cellular membranes and controls its clustering to membranes [121]. Furthermore, other PTMs have been shown to play a pivotal role in IFITM3 restriction of IAV. Indeed, phosphorylation of tyrosine 20 [122,123], ubiquitination of lysines 24, 83, 88 and 104 [104] and monomethylation of lysine 88 [122] have been shown to alter IFITM3 antiviral activity against IAV.

Importantly, in vivo studies in mice have confirmed that IFITM3 is a potent inhibitor of IAV infection [124,125]. Indeed, infection of *Ifitm3*^−/−^ mice with different strains of IAV leads to a fulminant viral pneumonia and to death. More specifically, upon infection with the low-pathogenic A/X-31 (H3N2) IAV strain, mice showed little difference in virus replication in the lungs during the first 48 h of infection. However, lungs of *Ifitm3^−/−^* mice contained 10-fold higher levels of replicating virus than the WT mice at 6 days post-infection and this was associated with profound morbidity [124]. Analysis of immune cells recruited to lungs showed that the lack of IFITM3 resulted in a reduced proportion of CD4+ and CD8+ T-cells but an elevated proportion of neutrophils [124]. This was also associated with increased levels of pro-inflammatory cytokines such as TNF-α, IL-6, G-CSF and MCP-1 [124]. Moreover, extrapulmonary lesions, such as myocarditis, have also been reported upon IAV infection in mice, and IFITM3 expression in cardiac tissue has been shown to protect mice against such cardiac lesions [126]. Interestingly, IFITM3 also plays a pivotal role in protecting immune cells from IAV infection. Following IAV infection in mice, IFITM3 is upregulated in respiratory DCs, limiting viral load and apoptosis in those cells. In the absence of IFITM3, DCs also show impaired migration from lung mucosa to the draining lymph node, thus preventing antigen presentation to naïve CD8+ T-cells and the establishment of adaptive immunity [127]. Similarly, IFITM3 is transiently expressed in activated T-lymphocytes, following cognate antigen recognition or type I IFN stimulation. However, IFITM3 is mostly expressed in lung tissue-resident memory T cells (T_RM_), a cell population that ensures the first line of defense against pathogen re-encounter while being directly exposed to infection. In T_RM_ cells, IFITM3 expression is maintained after primary IAV infection due to hypomethylation of its promoter, leading to enhanced survival after a new challenge with IAV [128]. Altogether, these observations indicate that IFITM3 plays a pivotal role in the establishment of both innate and adaptative immune responses.

As mentioned above, host genetics strongly impacts susceptibility to various infections. Two genetic variations within human *IFITM3* have been described to be associated with increased IAV infection severity. Both are single nucleotide polymorphisms (SNPs), the most prevalent being the rs12252-C, mainly present in Asian populations [129]. Multiple studies, including two meta-analyses, found that rs12252-C is associated with severe outcomes following IAV infection [124,130,131,132,133] (meta-analyses: [134,135]). However, these results are controversial, as other studies showed no association between the polymorphism and the risk of severe influenza [136,137,138]. rs12252-C is a synonymous SNP which is located at a splice acceptor site, potentially leading to a putative truncated IFITM3 with a 21 amino acid deletion at its N-terminus, termed D21-IFITM3 [124]. Nevertheless, an in vitro study showed that D21-IFITM3 still restricts IAV infection [139], and studies performed in rs12252-C homozygous patients have failed to detect the truncated isoform of IFITM3 by high-throughput RNA-sequencing [140,141]. The second SNP associated with severe risk of influenza infection is rs34481144-A, mainly present in Caucasian populations [129]. This SNP is present in the 5′UTR region of *IFITM3*, in the promoter region. It modulates IFITM3 expression by governing CTCF binding to the gene promoter, thus inactivating *IFITM3* transcription. However, in a recent Brazilian cohort, rs34481144-A was not associated with severity or mortality during IAV infection, nor with IFITM3 levels [138], highlighting the complexity of this type of analysis.

Considering the importance of several species in the zoonotic lifecycle of influenza viruses, numerous studies have elucidated roles of IFITM proteins in the restriction of IAV in avian species, swine and bats. Of note, the *IR-IFITM* gene cluster is conserved across mammals [142]. Indeed, it has been shown that five IFITM proteins are present in swine (two homologs of IFITM1, and one homolog of IFITM2, IFITM3 and IFITM5), which display antiviral activity against IAV in porcine cells [143]. The subcellular localization of swine IFITM proteins is similar to their human homologs [143]. In bats, IFITM3 has been shown to inhibit IAV cell entry and to be located in early/late endosomes [144]. Moreover, bat IFITM3 is S-palmitoylated on cysteines 71, 72 and 105, just as the human homolog, which is also important for its antiviral activity [145]. Finally, the repertoire of *IR-IFITM* is conserved between mammals and avian species. IFITM3 has been shown to restrict IAV in both chickens [146] and ducks [147], but no effect of duck IFITM1, IFITM2 and IFITM5 has been observed [147]. Following infection with both low and highly pathogenic avian influenza (LPAI/HPAI) strains, duck IFITM1/2/3 are highly upregulated in the lungs and the ileum from day 1 post-infection, whereas only IFITM3 was modestly upregulated in the ileum of chickens.

#### 4.1.2. NCOA7-AS: A V-ATPase Regulator, Preventing Endocytosis-Mediated Viral Entry

The short isoform of Nuclear Receptor Coactivator 7 (NCOA7), NCOA7-Alternative-Start (NCOA7-AS, also called NCOA7-B), belongs to the TLDc (Tre2/Bub2/Cdc16 (TBC), lysin motif (LysM), domain catalytic)-containing family of proteins. Seven TLDc proteins are found in humans and mice, including Oxidation resistance (OXR) proteins, and NCOA7 short and long isoforms (AS and Full-Length, FL, respectively) [148]. TLDc proteins have been shown to play a protective role against oxidative stress, through an unknown mechanism [149] (reviewed in [148]). NCOA7-FL is a known interactor of the estrogen receptor, which translocates to the nucleus upon estradiol treatment, where it was suggested to act as a transcriptional coregulator [150]. NCOA7-AS does not seem to share this property and is uniquely upregulated by type I IFNs via an internal promoter [151]. Recently, NCOA7-AS has been shown to inhibit infection with IAV and other viruses entering the cell via endocytosis, by limiting viral entry into the cytoplasm of host cells [152]. Both NCOA7-FL and -AS have been shown to interact with several subunits of the vacuolar ATPase (V-ATPase), the proton pump responsible for endolysosomal acidification [152,153,154]. Through an as yet unelucidated mechanism, NCOA7-AS interaction with the V-ATPase leads to a higher acidification of the endolysosomal system, which increases antigen degradation and seems detrimental to IAV by, possibly, affecting the ability of HA to allow fusion [152]. Interestingly, NCOA7-AS acts independently of IFITM3 [152], but further work is now warranted to fully elucidate the NCOA7-AS molecular mechanism of action as well as its importance in vivo.

#### 4.1.3. ZMPSTE24: An IFITM Cofactor, Which Also Acts Independently

Zinc-metallopeptidase STE24 (ZMPSTE24) is a zinc-metalloprotease containing seven transmembrane domains, which is constitutively expressed and localized at the membrane of cytoplasmic organelles as well as at the inner nuclear membrane [155]. In mammals, this protease is notably required for the maturation of lamin A, which is important for nuclear architecture. ZMPSTE24 has been recently identified as a restriction factor of a large number of viruses entering cells via endocytosis, among which IAV [155], [156]. However, ZMPSTE24 expression is not upregulated by type I IFNs or viral infection [155]. ZMPSTE24’s antiviral activity is independent of its protease activity, as shown by mutations of the conserved HEXXH catalytic motif [155]. Reminiscent of IFITM3 or NCOA7-AS, ZMPSTE24 inhibits cytosolic entry of IAV. Interestingly, ZMPSTE24 has been shown to interact with IFITM1/2/3 and to be required for IFITM antiviral activity. However, IFITM proteins are not necessary for ZMPSTE24′s antiviral activity, as overexpression of IFITM3 in *ZMPSTE24*^−/−^ cells does not restore the restriction phenotype. This suggests that ZMPSTE24 acts downstream of IFITM proteins, as well as independently of IFITMs. Furthermore, in vivo studies in laboratory mice revealed that deletion of *Zmpste24* leads to higher viral titers upon IAV infection, along with an increased production of pro-inflammatory cytokines and a higher mortality rate [155].

#### 4.1.4. CH25H: A Restriction Factor In Vitro but an Enhancer of Inflammation In Vivo

Cholesterol-25-hydrolase (CH25H) is an enzyme induced by type I IFNs and which catalyzes the oxidation of cholesterol into 25-hydroxycholesterol (25HC), a soluble oxysterol. Both CH25H and 25HC are important regulators of cholesterol homeostasis. Shortly, CH25H and 25HC inhibit the transcription factor sterol regulatory element-binding protein (SREBP) and activate the transcription factor liver X receptor (LXR). These two transcriptional factors have a great importance in modulating cellular cholesterol homeostasis [157]. Moreover, 25HC also represses the expression of the *HMGCR* gene and induces the degradation of the 3-hydroxy-3-methyl-glutaryl-coenzyme A reductase (HMGCR) protein, which leads to an inhibition of the cholesterol biosynthesis pathway. CH25H and its product 25HC have been shown to be involved in the restriction of a large number of enveloped viruses by inhibiting the fusion between viral and endosomal membranes [157]. This antiviral activity was also observed in vitro for IAV both in canine MDCK cells (i.e., the cell line typically used for IAV amplification *in vitro*) [158] and in murine immortalized airway epithelial cells [159], suggesting that CH25H is a restriction factor for IAV. However, surprisingly, deletion of *Ch25h* in a mouse model of IAV infection is protective [159], and is associated with a decreased inflammatory-induced pathology. This discrepancy between in vitro and in vivo results may be correlated with a role of CH25H as an amplifier of inflammation, which surpasses its restriction activity [159].

#### 4.1.5. B4GALNT2: A Factor with the Potential of Inhibiting Avian IAV Entry When Overexpressed

Genetic loss-of-function screens such as whole-genome CRISPR/Cas9 screens or RNAi screens have been widely used to identify host-dependency factors. However, little have focused on discovering factors that inhibit viral replication, until recently. Indeed, a new approach based on a whole-genome CRISPR/Cas9 activation screen recently led to the identification of β-1,4 N-acetylgalactosaminyltransferase 2 (B4GALNT2) as a pan-avian influenza virus inhibitor [160]. B4GALNT2 is a glycosyltransferase, which catalyzes the transfer of a N-acetylgalactosamine (GalNAc) to the penultimate galactose of α-2.3-linked sialic acids. Thus, without changing the relative amounts of sialic acids at the cell surface, B4GALNT2 prevents the interaction of HA with these receptors. The specificity of sialic acid targeted by this protein explains the spectrum of IAV strains inhibited, namely avian strains, which have a preference for α-2.3-linked sialic acids [160]. However, the importance of endogenous B4GALNT2 in the control of avian IAV has not been demonstrated by loss-of-function studies, which would be important to carry out. Moreover, as pigs are considered as mixing vessels, it would be interesting to study the role of B4GALNT2 in the inhibition of avian IAV replication in pigs.

In addition to B4GALNT2, this CRISPR/Cas9 activation screen identified two other potential influenza inhibitors: the transmembrane 9 superfamily member 2 (TM9SF2) and Ras Additionally, Rab Interactor 2 (RIN2) [160]. TM9SF2 is an endolysosomal protein whose role in the maturation of the endosome was postulated, while RIN2 is an interactor of the early endosome-associated protein Rab5. However, neither the molecular mechanisms involved in their restriction activity nor their physiological role have been further explored.

#### 4.1.6. MUC1: A Decoy for IAV Viral Particles

Airway epithelial cells secrete mucus, which constitutes one of the first physical barriers to infection in the lungs, acting like a shield blocking the binding of pathogens to their cellular receptors. Proteins from the mucin glycoprotein family, and notably cell surface mucins (cs-mucins), are important components of the mucus. They contain a large extracellular domain which displays several oligosaccharides, and which is non-covalently linked to a transmembrane domain, allowing it to be shed from the cell surface. Moreover, the cytoplasmic tail of cs-mucins can activate signaling cascades upon phosphorylation and interaction with intracellular adaptor molecules [161,162,163,164,165]. MUC1 is a member of the cs-mucin family, is constitutively expressed and is upregulated by type I IFNs [166]. In addition to its shield role, MUC1 was suggested to be an important downregulator of infection-driven inflammation responses upon bacterial infections [167]. Interestingly, this role extends to IAV infection, as IAV-infected *Muc1^−/−^* mice display, more rapidly, high viral loads and a faster, enhanced inflammatory response than wild-type mice [168]. In vitro, IAV was found to interact with MUC1 and to trigger the shedding of its extracellular domain [168]. This secreted extracellular domain could then act as a decoy and bind IAV to limit cell infections. As cs-mucins are highly conserved across species, it could be interesting to explore the potential antiviral role of MUC1 in other species.

### 4.2. Restriction Factors Inhibiting Viral Genome Replication

The steps of nuclear import, transcription, replication, protein translation, vRNP assembly and nuclear export are targeted by a large number of restriction factors acting either by inhibiting viral replication and transcription, translation or by degrading viral components such as the subunits of the viral polymerase or vRNAs.

#### 4.2.1. MX Dynamin-Like GTPases: Broad-Spectrum Antiviral Proteins with Possible Multiple Modes of Action

The family of large dynamin-like GTPases all share a similar three-dimensional structure and general organization: a N-terminal domain of various length and unknown structure, a globular head that sports the GTPase activity, a stalk domain as well as a middle domain (or bundle signaling element, BSE) that allows the flexibility of these mechanoenzymes [169]. Human Myxovirus resistance proteins 1 and 2, MX1 and MX2 (also named MxA and MxB) are members of this family and possess potent antiviral activity against a broad range of viruses [170]. MX1 is best known for its ability to inhibit IAV infection, but it can also inhibit a wide range of positive- or negative-single stranded or double stranded RNA viruses as well as certain DNA viruses [170], and MX2 inhibits HIV-1, Herpesviruses, HCV and HBV [171,172,173,174,175,176,177]. The MX genes were first discovered in mice (MmMx1), with the serendipitous observation that the A2G mice strain were resistant to IAV infection whereas most inbred laboratory strains, lacking a functional MmMx1 locus, were highly susceptible [178]. The subsequent creation of transgenic mice expressing a functional MmMx1 or human MX1 confirmed the importance in vivo of MX1 proteins [179,180]. MX1 is an ISG induced by type I or III IFN signaling [181], but it is commonly known that ectopic expression of MX1, without the induction of other ISGs, is sufficient for IAV restriction. Human MX1 is a cytoplasmic protein whereas mouse MmMx1 is mostly nuclear, which would probably hint at slightly different mechanisms of action for IAV restriction.

MX1 proteins possess intrinsic antiviral determinants that are essential for effective IAV restriction. Indeed, the presence of a functional GTPase domain [182], an intact BSE [183] and oligomerization via the stalk domain [184] are essential for correct anti-IAV activity. Other essential determinants are two loops localized at the end of the stalk: the L2 and L4 loops [185]. The L4 loop, which has been positively selected throughout evolution [186], harbors essential amino acids (F561 and G562) for its antiviral activity [187]. This is different from MX2, which only needs its N-terminal domain and an oligomerization domain for HIV-1 restriction [188,189,190]. Despite many years of intense study, the exact mechanism of action of MX proteins remains misunderstood. What is largely admitted nevertheless in the case of MX1 is that the main viral target in the case of IAV seems to be the viral NP protein, as it has been shown that mutations in NP can confer resistance to MX1 restriction [191,192,193,194]. This is reinforced by the fact that avian strains of IAV are more susceptible to inhibition than human strains and this has also been attributed to NP [180,195]. In the case of MmMx1, PB2 could also be an additional target [196,197]. MX1 proteins can form dimers and further oligomerize through numerous interfaces and have been theorized to form ring-like structures that could circle around vRNPs, possibly by interacting with NP (and potentially PB2) [196,198], but experimental data supporting this model are still lacking. In any case it seems that the antiviral mechanism of action of MX1 may act at several steps of the viral lifecycle, be it nuclear import of vRNPs, viral protein production or directly on replication itself [170,199,200,201]. To fully understand the fine details of MX1-induced IAV restriction, more studies are needed to confirm/contest the many hypotheses that exist in the literature.

Several studies have been performed to determine the importance of MX1 in humans through the search for polymorphisms within the human population [202,203,204]. G-G interface allelic variants were discovered, resulting in loss of GTPase activity, but were found in heterozygotes and did not show any dominant-negative effect on wild-type MX1 [202,203]. Several other variants were discovered in the stalk domain that resulted in loss of antiviral activity with dominant negative effects [203]. Unfortunately, no data of IAV infection history from homozygous carriers of these mutations are available. This aspect is nicely reviewed in [205].

Another interesting aspect of MX1 biology is the fact that mammalian MX1 proteins are active in cell types belonging to other species. For example, human MX1 expressed in mouse cells is active against IAV [206] and the opposite is also true for MmMx1 in human cells [196,207]. Transgenic mice expressing human MX1 are also protected against IAV infection [180,208,209] and have proven to be very useful models in the field. These observations may either suggest the existence of common crucial cofactors conserved between species, or that MX1 proteins are sufficient by themselves to inhibit IAV, but further in-depth studies are required to elucidate this.

A secondary, nonetheless important, role in IAV control was recently discovered for MX1. Indeed, MX1 was found to be an inflammasome sensor in human respiratory epithelial cells upon IAV infection, triggering IL-1β secretion [209]. The MX1 inflammasome formation was found to be dependent on MX1 oligomerization and also observed with cytoplasmic porcine MX1, but not nuclear MmMx1 [209], which may potentially be explained by the differential localization of these proteins. Transgenic mice expressing human MX1 induced inflammasome activation in respiratory epithelium upon IAV challenge [209], showing a potential relevance in vivo. Nevertheless, the authors showed that the MX1 inhibition of early steps of IAV replication was independent of the inflammasome [209], reinforcing the idea that MX1 plays multiple roles during IAV infection.

While MX1 from humans and mice as well as in other mammals such as rats, pigs and bats are potent anti-influenza A factors [210,211,212], in ducks and chickens, MX1 seems to be inactive against avian IAV. Indeed, chicken MX1 was shown to be devoid of antiviral activity against several low and highly pathogenic strains and to be non-essential for the IFN response in chickens [213,214,215,216]. This could be due to the fact that chicken MX1 does not possess a GTPase activity, which is known to be crucial for the anti-IAV activity of human and mouse MX1 [182,215]. Similar to chicken MX1, duck MX1 has not been found to possess antiviral activity against IAV [217]. It would be interesting to understand the molecular mechanisms that lead to this loss of function in poultry as MX1 seems to be a crucial IAV restriction factor, especially since the overexpression of human MX1 or MmMx1 in chicken cells is able to inhibit IAV replication [215].

#### 4.2.2. GBP Dynamin-Like GTPases: Indirect and Direct Inhibitors

Guanylate binding proteins (GBPs) are also part of the large dynamin-like GTPases, like MX proteins, and have been shown to have a broad antimicrobial restriction activity, encompassing bacteria, protozoa and viruses [218]. The GBP family is composed of seven members which are ISGs induced by type I and type II IFNs [219]. Some of these proteins have been shown to inhibit IAV [220,221,222,223]. Indeed, a splice variant of human GBP3, hGBP-3ΔC, and to a much lower extent GBP1, were shown to possess anti-IAV activity [220]. Overexpression of hGBP-3ΔC potently decreased IAV replication and, conversely, silencing of GBP3 significantly increased IAV replication (by over 1 log). The overexpression of hGBP-3ΔC decreased IAV polymerase activity and the accumulation of viral RNA species, and GTP binding was found to be necessary for IAV restriction by hGBP-3ΔC [220]. Another study observed antiviral activity of GBP1 through overexpression experiments [221], however depletion experiments are still lacking to confirm the physiological role of GBP1 in inhibiting IAV replication. GBP1 antiviral activity was suggested to be antagonized by NS1, but only ectopic expression of NS1 was used to show this [221], therefore further evidence is needed. In cells from guinea-pigs, guinea-pig GBP1 has been proposed to play a role in RIG-I mediated restriction of IAV, as its knockdown dampened the IFN response as shown by reducing the induction of MX1 mRNA after infection [224]. In line with this, ectopic expression and silencing experiments showed that human GBP5 modestly inhibits IAV replication, and this was proposed to be due to an indirect effect, through the regulation of the IFN and inflammatory responses [222]. Interestingly, GBP2 and GBP5 were also shown to inhibit the furin protease and therefore to have a negative impact on infection by various viruses, including a modest effect on IAV [223]. On the contrary, GBP7 knockout out by CRISPR/Cas9 reduced IAV replication and its overexpression increased IAV replication [225]. This was found to be due to the suppression of the innate immune system by GBP7, defining it as a proviral factor [225]. In the future, it would be interesting to see if all these different observations could be recapitulated in *Gbp* KO mice. Finally, non-synonymous polymorphisms in human GBP2 have been linked to a reduced responsiveness to IFN treatment during HBV treatment [226], but no data on the relevance of GBP polymorphisms in IAV infection response have been reported.

#### 4.2.3. TRIM Proteins: A Large Family of Antiviral Proteins Implicated in Innate Immune Signaling

The tripartite motif (TRIM) protein family contains more than 80 proteins in humans, which are involved in various cellular processes, including cell proliferation, autophagy and immunity [227]. They are characterized by a conserved domain organization at the N terminus (known as the TRIM or RBCC motif) composed of a catalytic RING domain which possesses an E3-ubiquitin ligase activity, one or two B-box domains and a coiled-coil dimerization (CCD) domain. They also contain C-terminal domains, which vary among TRIM proteins, for instance the SPRY domain or the PRY domain [227]. Various TRIM proteins have been shown to restrict IAV infection and act either by inducing the degradation of viral components or by inhibiting viral replication.

- Role of TRIM22, TRIM41 and TRIM14 in the degradation of viral components:

TRIM22 has been shown to be upregulated by type I IFNs [228,229] or by IAV infection and to inhibit IAV [228]. *TRIM22* knockdown increases IAV replication by more than one order of magnitude at low multiplicities of infection [228]. Moreover, TRIM22 is one of the players of the IFN-induced restriction of IAV as shown by the reduced inhibition observed once silenced [228]. Overexpression experiments have shown that TRIM22 interacts with NP and poly-ubiquitinates it via its RING domain, leading to NP degradation in a proteasome-dependent manner [228]. Interestingly, some H1N1 strains such as the H1N1 strains isolated in 1933 and 1934 (i.e., WSN and PR8) and pH1N1 were shown to be insensitive to TRIM22-mediated antiviral activity, whereas more recent seasonal H1N1 isolates were restricted by TRIM22 [230]. The presence of four R to K substitutions in NP differentiate the restricted strains from the non-restricted, potentially being the consequences of adaptation due to a sustained circulation in humans since 1918. Introduction of these mutations in resistant strains sensitizes them to TRIM22 restriction. This showed that adaptative mutations may sometimes surprisingly induce greater sensitivity to innate immune effectors [230].

Additional TRIM proteins inhibit IAV by interacting with NP, promoting its degradation. This is the case of TRIM41, which is notably not IFN-inducible [229,231]. TRIM41 interacts with NP via its C-terminal SPRY domain, mediates NP poly-ubiquitination via its RING domain, which in turn induces NP degradation by the proteasome [232]. Interestingly, TRIM14, a TRIM protein lacking a RING domain and upregulated by type I IFNs [229], has also been proposed to restrict IAV and to interact with NP [231]. This interaction, notably mediated by the PRYSPRY domain, alters NP stability by K48-linked polyubiquitination and NP-mediated proteasomal degradation. This suggests that the NP-TRIM14 interaction leads to the recruitment of a yet-to-define functional E3 ubiquitin ligase to mediate NP polyubiquitination [231].

TRIM32 was identified as a PB1 binding partner by mass spectrometry [233]. TRIM32 is constitutively expressed and located in the cytoplasm in normal conditions, but translocates to the nucleus upon IAV infection. TRIM32 overexpression inhibits IAV infection and its silencing increases IAV replication (by ~1 log) as measured by plaque assays. TRIM32 seems to inhibit IAV infection by inducing poly-ubiquitination of PB1, which leads to its proteasomal degradation and to the inhibition of the viral polymerase activity [233]. Further studies are still warranted to demonstrate the physiological role of TRIM32 in the control of IAV replication by using *Trim32*-deficient mice [234,235].

Finally, TRIM35, another ISG, which plays a general role in the induction of type I IFNs, leads to PB2 K48-linked polyubiquitination and to its proteasomal degradation [236]. Importantly, *TRIM35* silencing has a significant impact on IAV replication in vitro (about 1 log) and *Trim35^−/−^* mice are more susceptible to IAV replication and induced death [236].

- Role of TRIM25 and TRIM56 in the inhibition of viral genome replication:

TRIM25 is a protein that plays a fundamental role in RIG-I-dependent innate immune sensing of IAV and other viruses. Indeed, ubiquitination of RIG-I by the E3 ubiquitin ligase TRIM25 is necessary for RIG-I activation, thus leading to the production of type I and III IFNs following recognition of RNA ligands (reviewed in [237]). Interestingly, the NS1 proteins from all IAV strains can interact with TRIM25, however this interaction does not always translate to an inhibition of the IFN response [84,85,86]. Interestingly, an additional role of TRIM25 in the restriction of IAV has been recently unraveled [238]. Indeed, nuclear TRIM25 interacts with vRNPs and inhibits viral RNA synthesis independently of its ubiquitin ligase activity. More precisely, TRIM25 inhibits the onset of RNA elongation by preventing the movement of viral RNAs into the RdRp [238].

The overexpression of another TRIM protein, TRIM56, has been shown to restrict IAV infection (by 1 log) as measured by plaque assays [239]. Moreover, stable knockdown of *TRIM56* led to a 6-fold increase of de novo infectious virion production. Interestingly, TRIM56 antiviral activity is independent of its ubiquitin ligase activity and overexpression of a 63-residue segment present in its C-terminal has the same antiviral activity as the full length TRIM56. Moreover, the authors showed that viral RNA synthesis was impeded in infected cells overexpressing TRIM56 [239]. However, an in vivo study demonstrated that *Trim56^−/−^* were not more susceptible to IAV infection than wild type mice, failing to validate a physiological role of TRIM56 in the control of IAV replication in vivo [240].

Of note, the validation of the antiviral activity of some of the TRIM proteins in vivo can be challenging, not because of the absence of mice models, but given the importance of many TRIM proteins in the activation or regulation of innate immune sensing pathways. Moreover, TRIM proteins have not been extensively studied in pigs, bats or birds in terms of diversity of the repertoire, conservation and antiviral activity compared to their human orthologs, studies which could nevertheless be of great interest.

#### 4.2.4. ZAP and ZFP36L1: Antiviral Zinc Finger Proteins

Zinc finger Antiviral Protein (ZAP, also named ZC3HAV1 for Zinc Finger CCCH-type containing Antiviral 1) is an IFN-induced antiviral protein that inhibits a large variety of viruses (reviewed in [241]). ZAP is expressed as four isoforms (S, M, L and XL) [242]. However, ZAP-S and ZAP-L are the predominantly expressed isoforms. ZAP-S and ZAP-M isoforms are upregulated by type I IFNs, ZAP-S being the most upregulated [242]. The main two isoforms of human ZAP protein (ZAP-S and ZAP-L), arising from alternative splicing, differ only by the presence or the absence of a poly ADP-ribose polymerase (PARP)-like domain at the C-terminus. Both isoforms have a N-terminal domain containing four CCCH-type zinc fingers as well as a large central domain containing a TiPARP homology domain (TPH) and a WWE domain. The four zinc fingers are responsible for the RNA binding [243]. A zinc ion is coordinated via three cysteines and one histidine in each zinc finger, although their folding and protein sequences are different.

ZAP has been shown to inhibit viral replication by repressing the translation or promoting the degradation of viral mRNAs. To mediate this antiviral activity, ZAP recognizes and interacts directly with specific RNA elements enriched in CpG dinucleotides [243,244]. ZAP-L silencing modestly increased IAV replication in human cells, as measured by plaque assays [245]. Ectopic expression of tagged viral proteins and ZAP-L showed that ZAP-L interacts with PB2 and PA subunits of IAV polymerase in a PARP domain-dependent manner [245]. ADP-ribosylated PA and PB2 are associated with ZAP-L and then ubiquitinated, which leads to their proteasomal degradation. The viral protein PB1 interacts with ZAP-L and counteracts its antiviral activity. Indeed, PA and PB2 binding sites on ZAP-L are in close proximity to their PB1 binding sites, thus leading to the dissociation of PA and PB2 from ZAP-L [245]. Furthermore, the ability of ZAP-S (which lacks the PARP domain) to inhibit IAV has also been shown by overexpression experiments [246]. However, the relevance of these findings is questionable, as *Zc3hav1* knockout had no impact on IAV replication in murine embryonic fibroblasts (MEFs) [246]. ZAP-S overexpression inhibits viral protein expression by binding to viral mRNAs, promoting their degradation and inhibiting their translation [246]. The viral protein NS1 seems able to antagonize overexpressed ZAP-S through an unknown mechanism [246]. Nevertheless, it is tempting to speculate that NS1 might antagonize ZAP-S through TRIM25 inhibition given that the TRIM25-mediated ubiquitination of ZAP-S seems critical for its antiviral activity and that some NS1 mutants fail to do so [246]. No current validation of ZAP importance in vivo to restrict IAV replication has been published. However, *Zc3hav1^−/−^* mice have been previously described in the literature and could be used to serve that purpose [247,248].

*ZAP* appears to be evolutionary conserved in animals [249,250,251]. However, little is known about the antiviral activity of *ZAP* orthologues in pigs, bats or in avian species. Analysis of the antiviral activity and the binding specificity of ZAP from different avian species, showed that ZAP from aquatic birds exhibited a broader antiviral activity, potentially due to a lesser selectivity for CG-enriched RNA elements [250]. It could be of great interest to analyze the restriction of IAV by ZAP from different species, given that ZAP appears to be an important selection pressure shaping genome composition and could greatly influence cross-species transmission.

Another CCCH-type zinc finger protein, ZFP36L1 has been recently discovered to harbor anti-influenza activity [252]. ZFP36L1 was shown to be induced upon TNF-α treatment and by viral infection suggesting a potential role of this protein in the host cell antiviral defense. However, ZFP36L1 is not IFN-inducible [166]. Overexpression of ZFP36L1 decreased viral titer (around 1 log), as measured by plaque assays and decreased M1, M2, NS1, NS2 and HA protein levels, but not mRNA levels. Conversely, knockdown of this protein increased viral replication (around 1 log), and this was rescued by complementation. The proposed mechanism of action is that ZFP36L1 decreases M1 and NS2 protein levels, inhibiting vRNP nuclear export. This is supported by the fact that NP seemed to be trapped in the nucleus under overexpression conditions. *Zfp36l1^−/−^* mice have been generated in other studies [253] and could be used to validate this restriction factor in vivo.

#### 4.2.5. IFITs: Friends or Foes?

The IFN-induced proteins with tetratricopeptide repeats (IFITs) are an RNA binding family of ISGs that are among the most upregulated during antiviral signaling. There are four IFIT proteins coded in humans: IFIT1 (or ISG56), IFIT2 (or ISG54), IFIT3 (or ISG60) and IFIT5 (or ISG58). These proteins are known to inhibit a wide range of viruses [254]. The first antiviral activity attributed to these proteins was the ability to bind eIF3 subunits and decrease cap-dependent translation efficiency [255]. Later, IFITs were found to bind non-self 5′-ppp or 2′-O unmethylated RNA [256,257,258] as well as to viral proteins [259]. Additionally, of note is the fact that IFITs can associate with each other which can modulate their functions (reviewed in [260]). In the case of IAV, data is quite conflicting. Indeed, the individual silencing of *IFIT1*, *2* and *3* was reported to increase IAV replication, as measured by a viral PolI reporter expression system after infection, suggesting the IFIT1/2/3 heterotrimer complex could be responsible for this antiviral activity [256]. However, interestingly, another more recent study, using both CRISPR/Cas9 knockout and overexpression experiments, showed that human and murine IFIT1 are not major restriction factors of IAV [261]. The authors also showed that IFIT1 actually has a low binding affinity for 5′-ppp. Moreover, in vivo studies have shown that *Ifit1^−/−^* mice do not show any differences compared to WT mice in regard to IAV infection, indicating that murine IFIT1 does not have a major role in restriction or pathogenesis of IAV in vivo [261]. In addition, a major study recently showed that IFIT2 is actually a cofactor for IAV [262]. Indeed, IFIT2 was shown to be repurposed by IAV to become a proviral effector promoting the translation of viral mRNAs [262]. Therefore, further studies are required to understand the role, if any, of mammalian IFITs as restriction factors of IAV in vitro and in vivo.

An avian IFIT (avIFIT) protein in ducks was found to resemble the structure of mammalian IFIT5. Upon testing, the authors showed that chicken cells stably expressing duck IFIT inhibited IAV infection (by 1 log) [263]. They also showed that avIFIT was able to bind to NP and also enhance the IFN response. Chicken IFIT5 (chIFIT5) may also harbor antiviral properties as it has been shown to be able to interact with 5′-ppp-containing viral RNAs [264]. The same group later showed that transgenic chickens stably expressing chIFIT5 had marked resistance to H5N1 infection [265], while another study showed that CRISPR/Cas9 chIFIT KO cells allowed for higher IAV replication (around 1 log) [263]. Further studies are needed to understand the molecular mechanisms at play for avian IFIT IAV inhibition and the differences between the avian and mammalian proteins.

#### 4.2.6. OAS-Family Proteins: NS1-Counteracted dsRNA Binding Inhibitors

Humans possess four genes coding for the 2′,5′-oligoadenylate (2-5A) synthetase (OAS) proteins: OAS1, 2, 3 and OAS-like (OASL), that are IFN-inducible [166,266]. Once activated by dsRNA, OAS1, 2 and 3 are able to synthesize 2-5A which bind to monomeric RNaseL, inducing its dimerization and activation. Active RNaseL then cleaves cellular or viral ssRNA and can induce autophagy and apoptosis, which is detrimental to viral replication [267]. The small RNAs resulting from RNaseL cleavage of ssRNA have been also shown to activate RIG-I signaling, leading to IFN production [268,269]. OASL lacks the ability to produce 2-5A, but has nevertheless been shown to enhance RIG-I sensing [270].

The OAS/RNaseL system is able to inhibit a wide number of RNA viruses [271], but in the case of IAV, it has been shown that NS1 protects from the effects of this pathway [272,273]. Indeed, a virus encoding a dsRNA-binding deficient NS1 protein was sensitive to the RNaseL pathway [272], suggesting that NS1 dsRNA binding capability counteracts OAS dsRNA recognition, preventing RNaseL activation and subsequent ssRNA degradation. OAS3 has been shown to have a higher affinity for dsRNA compared to OAS1 and OAS2 and also to be the main producer of 2-5A in IAVΔNS1 infected cells, as *OAS3* KO abolished 2-5A production and raised viral titer [273]. The importance of OAS proteins in vivo has not been studied (of note, *Rnasel*^−/−^, but not *Oas*^−/−^ mice exist [274]), but they are likely to have little impact seeing as OAS proteins are antagonized by NS1.

#### 4.2.7. PKR and NF90: An Example of Host–Virus Coevolution

Protein kinase R (PKR) is a serine/threonine kinase that possesses two N-terminal dsRNA-binding motifs as well as an effector C-terminal kinase domain [275]. PKR is constitutively expressed and slightly upregulated by IFN treatment [166]. During viral infection, PKR recognizes dsRNA which induces its dimerization and the subsequent phosphorylation of the eukaryotic translation initiation factor 2 (eIF2α) at position serine 51 [276]. This has the consequence of shutting down cellular translation, therefore inhibiting the replication of numerous viruses [277]. However, IAV seems to have evolved countermeasures. Indeed, in IAV infected cells, there is an increase of P58IPK which can inhibit PKR, thereby promoting viral protein production and efficient replication [278]. NS1 has also been shown to play a protective role against PKR by binding to viral dsRNA blocking recognition by PKR [279,280,281,282]. In line with this, IAV with defective or missing NS1 are more susceptible to the effects of PKR [279,280,281]. NS1 mediated inhibition of PKR has been mapped to two essential residues in the NS1 N-terminal domain: R35 and R46 [282]. Of note, mice lacking PKR are more susceptible to IAV infection, suggesting a role in vivo for this antiviral protein [280,283].

In chickens, PKR expression is induced following H5N1 inoculation, but this induction is not sufficient to induce resistance [216]. Mallard duck’s PKR anti-IAV phenotype has yet to be studied, but it has been shown to be upregulated during IAV infection and to miss the N-terminal second RNA binding domain compared to human PKR, which could affect its function [284].

Interestingly, a cellular countermeasure to the NS1-mediated PKR inhibition has recently been discovered: the protein Nuclear factor 90 (NF90), another dsRNA-binding protein [285,286] able to inhibit different viruses [287]. NF90 does not seem to be induced by IFN [166]. NF90 interacts with both NS1 and PKR, reducing the impact of NS1′s PKR inhibition [288]. This is an interesting example of coevolution between viruses and host cells. NF90 was also shown to exert antiviral activity against NS1-deficient IAV by interacting with the C-terminus of PKR and upregulating PKR phosphorylation (knockdown experiments in 293T cells) [289]. Of note, knockdown and overexpression experiments showed that NF90 also independently but modestly inhibits IAV by interfering with viral polymerase activity, possibly through an interaction with NP [290,291]. Unfortunately, *Nf90^−/−^* mice do not seem to be viable [292], so exploring the extent of NF90 importance in IAV infection in vivo will be complicated.

#### 4.2.8. MOV10 and DDX21: Antiviral Cellular Helicases

Helicases are enzymes that unwind double-stranded nucleic acids, and are classified into three super-families and two small families based on conserved motifs (reviewed in [293]). Some of these helicases have proven to possess antiviral activity against many different viruses [294]. Here, we describe two helicases that have been shown to inhibit IAV.

Moloney leukemia virus 10 (MOV10) is a 5′ to 3′ RNA helicase from the UPF1-like family [295]. It has been shown to play roles in miRNA metabolism, mRNA stabilization and translation, retroelement inhibition and viral restriction [296,297,298,299] and is partially upregulated by type I and II interferon treatment [166], [300]. MOV10 has also been proposed to be an IAV restriction factor. Indeed, MOV10 was identified by mass spectrometry as an IAV vRNP binding partner, through an interaction with NP [301]. *MOV10* silencing increased IAV replication (by ~1 log) and conversely, MOV10 overexpression decreased IAV replication [301,302]. MOV10 seems to inhibit IAV polymerase activity by preventing NP nuclear import [301,302] and by possibly sequestering incoming vRNPs inside P-Bodies [302]. Of note, NS1 was proposed to counteract MOV10 activity [302]. MOV10 has also been shown to inhibit other viruses by enhancing the production of IFN in a RIG-I/MAVS independent manner [300], and it would be interesting to determine whether this indirect activity play also a role in IAV inhibition. *Mov10^−/−^* mice are not viable [303], rendering difficult the in vivo investigation of the importance of this protein in IAV infection control.

DDX21 is part of the DEAD (Asp–Glu–Ala–Asp)-box family of RNA helicases which are RNA-binding helicases that play fundamental roles in RNA metabolism [304]. DDX21 has been shown to resolve R loops (i.e., DNA:RNA hybrids), promoting genomic stability [305]. It has also been shown to be slightly upregulated by type II IFN [166] and to harbor antiviral activity against RNA viruses [306]. *DDX21* silencing increases IAV replication in vitro as shown by plaque assays [307]. DDX21 binds to PB1, preventing the assembly of the polymerase, thus inhibiting replication [307]. However, NS1 is able to attenuate this restriction by binding DDX21 and suppressing its antiviral activity [307]. Its physiological importance has not been demonstrated yet due to the lack of *Ddx21^−/−^* mice.

#### 4.2.9. HDACs: Indirect Inhibitors

Histone Deacetylases (HDACs) are involved in the deacetylation of acetylated proteins [308]. These proteins are classified into four classes: class I, class II, class III and class IV HDACs, with only some of the members being upregulated after IFN treatment, such as HDAC4 and HDAC6 with type II IFN [166]. Recent publications have suggested roles for different members of this family in IAV replication, either as dependency factors or as inhibitors.

Silencing experiments showed that HDAC1, a class I HDAC, modestly increases viral infection, whereas HDAC8 seems to act as a viral cofactor [309,310]. The modest anti-IAV effect of HDAC1 was linked to STAT1 phosphorylation regulation and ISG induction [310]. A similar role was reported for HDAC2 [311].

Concerning class II HDACs, HDAC6 was proposed to be essential for IAV uncoating [312], however, *HDAC6* silencing has an overall positive impact on IAV replication as measured by plaque assays [313]. Pharmacological inhibition of HDAC6 increased trafficking of viral components to the plasma membrane and viral release, a mechanism probably involving acetylated microtubules [313]. More recently, *Hdac6*^−/−^ mice were shown to be slightly more susceptible to influenza infection, which was actually correlated with a downregulation of several innate immune pathways [314]. Further clarification on the impact and the role of HDAC6 is therefore required. Another class II HDAC, HDAC4, has also been proposed to harbor a modest anti-influenza activity [315].

Class III HDACs, also known as sirtuins, have been proposed to modestly inhibit influenza virus infection through depletion experiments, without further characterization [316].

Finally, a class IV HDAC member, HDAC11, has also been shown to exhibit anti-influenza activity, probably by regulation of the IFN response [317].

The roles of HDACs in the host cell influenza defense are often modest and indirect, with some members playing proviral rather than antiviral roles. More studies would therefore be necessary to understand the molecular mechanisms and the importance in vivo of HDACs on IAV infection.

#### 4.2.10. ANP32s: Major Cofactors and Potential Minor Inhibitors

The mammalian acidic nuclear phosphoprotein 32kDa (ANP32) family consists of five members: ANP32A, B, C, D and E, but there is an ongoing debate over the true existence of C and D as *bone fide* expressed genes. These proteins all contain an N-terminal leucine-rich repeat (LRR) region, a central region and a C-terminal unstructured low-complexity acidic region (LCAR) region. ANP32 proteins are known to carry out various roles such as chromatin regulation, phosphatase regulation, involvement in apoptosis and intracellular transport [318]. ANP32A was initially discovered to be a major co-factor of IAV determining species specificity [319], mediating the assembly of the IAV replicase [320].

Identification of ANP32A as a major factor in IAV life cycle and in host restriction/adaptation was a breakthrough. It was known for a long time that the polymerase from classical avian influenza A viruses did not function efficiently in human cells and that the PB2 subunit, and in particular residue 627, contributed to this host restriction [321,322]. The lack of a cellular cofactor for the avian polymerase in mammalian cells was previously suggested to be responsible for this restriction [323]. Chicken ANP32A was indeed identified through an elegant genetic screen as an essential cofactor allowing an avian-origin PB2 protein to function in mammalian cells [319]. In addition, ANP32A was shown to be crucial for IAV replication in both birds and mammals, but avian-adapted polymerases are not able to efficiently use mammalian ANP32A. A 33 amino acid motif is missing from the mammalian version of ANP32A in comparison to avian ANP32A proteins and introduction of this motif in mammalian ANP32A allows avian-origin PB2 to function in mammalian cells [319]. Interestingly, ostriches and other ratites lack this region, which elegantly explains the previous observation that ostriches behave more like mammalian species regarding avian IAV PB2 adaptation (i.e., acquisition of the mammalian signature PB2 E627K) [324]. Later, both ANP32A and ANP32B were shown to be essential for IAV replication in human cells, but either of them being redundant, as cells that lacked one were still able to support the polymerase, but cells lacking both could not [325].

Another study, which confirmed the importance of ANP32A and ANP32B as host cofactors, highlighted that other members of ANP32 family such as ANP32C, ANP32D and ANP32E have an opposite, negative effect on IAV replication [326]. Indeed, overexpressing human ANP32C or ANP32D together with ANP32A had a negative effect on the replication of IAV (possessing either the avian PB2-627E signature or the human PB2-627K signature) compared to ANP32A overexpression alone [326]. ANP32E only had a negative effect for avian PB2-627E signature IAV, but knockdown of *ANP32E* did not show any effect [326]. Of note, *Anp32e^−/−^* mice have been produced and are viable [327], but seeing as a mice KO model of ANP32A did not show any significant effect on IAV infection [328] we could imagine a similar outcome for *Anp32e^−/−^* mice. Nevertheless, more studies must be performed to assess the mechanisms through which ANP32C, ANP32D and ANP32E might impair viral polymerase activity.

Chickens also possess ANP32s but with the particularity that chicken ANP32A contains a splicing site, which allows for the production of two splice variants, ANP32A-33 and ANP32A-29, of which ANP32A-33 is more potent [329,330]. A team recently made the discovery that SRSF10 is able to bind to a cis-regulatory element, promoting the production of ANP32A-29 transcripts and decreased that of ANP32A-33 transcripts. This had a negative impact on avian IAV polymerase activity but not mammalian strains, as mammals do not produce the two ANP32A-29 and ANP32A-33 splice variants [331].

#### 4.2.11. ISG15: ISGylation-Mediated Inhibition of NS1 Activities

Interferon-stimulated gene product 15 (ISG15) is one of the most upregulated ISGs [332]. It is a ubiquitin-like protein known to be conjugated to lysine residues of proteins in a process called ISGylation. This process involves the E1 activating enzyme Ube1L, E2 conjugating enzyme UbcH8 and E3 ligase enzymes Herc5 or TRIM25/EFP. The effects on cellular proteins of ISGylation are many (reviewed in [333]), such as the enhancement of antiviral signaling through IRF3 ISGylation to give an example [334]. Many viral proteins have been discovered as targets of ISGylation [333]. The first evidence of ISG15 impacting IAV replication was that *Isg15* deficient mice were shown to display increased susceptibility to IAV infection [335,336]. It is thought that NS1 is the target of ISG15′s antiviral activity, as NS1 has been shown to be a target of ISGylation, which prevents a number of its essential functions [337,338]. Further studies on ISG15 are therefore required to fully understand its impact on IAV infection.

#### 4.2.12. ISG20: An Exonuclease Affecting IAV Replication When Overexpressed

IFN-stimulated gene 20 kDa protein (ISG20) is a 3′ to 5′ exonuclease with specificity for ssRNA and is induced by interferon signaling [339]. This protein has been shown to inhibit a wide range of viruses, including IAV [340,341]. ISG20 overexpression was shown to decrease IAV replication [342,343], and an IFN-inducible, long-non-coding RNA sharing most of its sequence with ISG20, Lnc-ISG20, and regulating ISG20 expression, was shown to also impact IAV replication [344]. The effect of overexpressed ISG20 was shown to be dependent on its exonuclease activity and attributed to an interaction with NP, as shown by immunoprecipitation using overexpressed ISG20 and viral infection [343]. Although not confirmed for IAV, the antiviral effect of ISG20 against some viruses has also been shown to be attributed to an upregulation of the type I IFN response [345] or to an inhibition of viral translation [341]. Evidence of a physiological role of ISG20 against IAV, using depletion experiments, as well as in vivo experiments are still lacking. Of note, *Isg20^−/−^* mice are available [341] and could be used to this end.

### 4.3. Restriction Factors Affecting Viral Assembly, Egress or Maturation

To date, only a few host restriction factors have been shown to interfere with the late stages of IAV replication cycle. The late stages comprise viral assembly, egress and maturation of newly generated virions.

#### 4.3.1. Tetherin/BST-2: A General Inhibitor of Viral Release with a Controversial Activity on IAV

Bone marrow stromal antigen 2 (BST-2), also known as Tetherin, is encoded by an ISG [346]. This protein is a type II transmembrane protein with a unique topology, possessing a N-terminal transmembrane region, an ectodomain and a C-terminal glycosylphosphatidylinositol (GPI) anchor [346]. In humans, BST-2 is located at the trans-Golgi network (TGN), in endosomes and within lipid rafts. Its name, Tetherin, was given due to its ability to restrict viral release, by retaining viruses at the cell membrane like an anchor [347,348]. However, the anti-IAV activity of Tetherin remains quite controversial. Indeed, some studies describe an inhibition of IAV (or of IAV-derived virus-like particles) egress, [349,350,351,352,353], while some others refute an inhibitory role [354,355,356,357]. Interestingly, not all IAV strains could be restricted by Tetherin, suggesting a strain-specificity that could, in part, explain the controversial results concerning its restricting activity [353,358]. The inhibitory activity of Tetherin was proposed to be counteracted by some viral proteins, like M2 [356] or NA [354,355,358], but a role of M2 was also excluded [350]. Finally, in vivo studies performed on *Bst2*-deficient mice revealed that they were not more susceptible to IAV infection than wild-type mice, which would be in favor of the absence of antiviral activity of Tetherin, at least against the strain used in this study [351]. Further studies, using other strains, would be required to definitely conclude the absence of impact of Tetherin on IAV replication in vivo.

Orthologs of *BST-2* are present in a high number of species, including mammalian species, but also birds, alligators, turtles and some fish [359]. Even if there is often no amino acid sequence homology between the orthologs from different species, they share the same unique topology. Only a few of these orthologs were tested for their ability to restrict influenza viruses. Chicken encodes an ortholog of BST-2 (chBST-2) [360,361] and to date, chBST-2 antiviral activity was described and characterized for only one virus, the avian sarcoma and leukosis virus (ASLV) [361]. Further studies are required to determine if IAV is restricted by chBST-2.

#### 4.3.2. Viperin: An IAV Inhibitor When Overexpressed

Virus inhibitory protein, endoplasmic reticulum-associated, IFN inducible (viperin) is an ISG that was shown to inhibit a wide range of viruses through diverse mechanisms [362,363,364,365]. The first evidence for viperin inhibiting IAV was through overexpression experiments that showed that the inhibition was by disrupting lipid rafts in vitro, therefore inhibiting viral release [366]. Further studies confirmed an antiviral phenotype through overexpression in vitro [354,367]. However, *Viperin*^−/−^ mice did not show an enhanced viral load or lung damage, implying that viperin does not have an anti-IAV role in vivo [367].

#### 4.3.3. PAI-1: An Inhibitory Protein Having an Effect in the Extracellular Media

Plasminogen activator inhibitor-1 (PAI-1) is part of the serine protease inhibitor (SERPIN) family and is coded by the *SERPINE1* gene. The main role of this protein is to regulate the activation of plasminogen, the inactive precursor of plasmin, a serine protease involved in clearing of fibrin blood clots, by inhibiting both the urokinase-type plasminogen activator (u-PA) and the tissue-type plasminogen activator (t-PA) [368]. A surprising role for this protein in the inhibition of IAV maturation was recently discovered. Indeed, an image-based screen to identify novel ISGs regulating the late stages of IAV infection led to the identification of secreted PAI-1 as able to inhibit glycoprotein cleavage therefore reducing the infectivity of newly produced IAV particles [369]. *Serpine1^−/−^* mice presented a slightly increased susceptibility to IAV infection as well as a more severe disease phenotype. Interestingly, human genetic variability in *SERPINE1* may also influence virus spread and disease severity [369]. PAI-1 is therefore the first ISG to be described as having an inhibitory role against IAV in the extracellular media.

### 4.4. Additional Factors Inhibiting IAV

Other proteins have been shown to inhibit IAV, but very little is currently known with respect to their potential mechanism of action. A few of these factors are listed in the following section.

Phospholipid scramblase 1 (PLSCR1) has been shown to play a role in the inhibition of a number of viruses [370,371]. For IAV, a single study has suggested that overexpressed PLSCR1 can inhibit IAV replication (by 2 log) and that KO increased it (by 1 log) [372]. Overexpressed PLSCR1 can form a complex with NP and members of the importin α family and its proposed mechanism of action would be the inhibition of NP entry into the nucleus [372].

The knock-out of SERTA domain containing 3 (SERTAD3), an IFN-inducible transcription factor, was shown to have a 3 to 4-fold positive impact on IAV replication in human cells, and, conversely, its overexpression negatively affected replication [373]. *Sertad3^−/−^* mice were also somewhat more susceptible to IAV infection [373]. The potential mechanism of inhibition of SERTAD3 was suggested to be the inhibition of the polymerase as overexpressed SERTAD3 interacted with PA, PB1 and PB2, preventing their assembly [373].

Eukaryotic translation elongation factor 1 delta (eEF1D) is a subunit of the eEF1 complex, which is involved in translation elongation and other non-canonical processes. *eEF1D* knock-out has been shown to improve IAV replication (by 1 log) and, conversely, its overexpression caused a decrease in replication (by 1 log) [374]. This has been linked to an interaction between eEF1D and vRNP components, leading to a decrease in nuclear import, rather than its effect on viral translation as could have been expected [374,375].

Finally, the desmosome component Plakophilin 2 (PKP2) was discovered through an IAV comparative interatomic study to be a potential PB1 interactor [156]. Overexpressed PKP2 decreased infection (by 3- to 4-fold) and siRNA experiments increased infection levels (by 2- to 3-fold) [156]. The suggested mechanism of action is through the disruption the IAV polymerase complex by competing with PB2 [156].

## 5. Conclusions and Perspectives

As we have seen throughout this review, host cells possess a wide array of restriction factors, belonging to different families of proteins, that can act as one of the first barriers of the intrinsic and innate immunity against influenza virus infection. These antiviral proteins target multiple steps of the viral life cycle, be it entry, replication, maturation, assembly, egress or even have an effect in the extracellular media once the virus has budded. Type I and III IFNs are important players in the innate immunity to control IAV replication in vivo in humans, avian species and mice. In other mammalian species such as pigs and bats, this importance has only been shown in vitro. The protection induced by type I/III IFNs is due to the expression of several hundreds of ISGs. Indeed, the vast majority of the antiviral proteins described in this review are induced by type I and III IFNs, showing the strong relationship between these factors and the IFN response. However, in the host–virus arms race and in order to overcome the antagonism of the IFN response, viruses have evolved proteins, such as NS1, PB1-F2 or PA-X, to counteract either the induction of the IFN response or to directly inhibit the restriction factors (e.g., the NS1-induced inhibition of PKR) and, thus, to promote viral replication.

Despite extensive studies and decades of investigation on some anti-IAV factors, such as MX1 or IFITM3, mechanistic details are still lacking to fully understand the restriction they impose on IAV. Moreover, the importance of most restriction factors in vivo or their role in the adaptive immunity are still unknown or debated for the vast majority. Given that some restriction factors play a role in both antiviral restriction and induction/regulation of immune responses, results obtained in KO mice might be challenging in their interpretation. Of note, the type I/III IFN response is important for the innate immunity but also crucially shapes adaptative immune responses. In line with this, recent studies have shown that some of these factors such as IFITM3 are versatile actors at the interplay between innate and adaptive immunity. Indeed, these antiviral factors seem to modulate dendritic cells and antiviral CD4+ and CD8+ T cell-responses. However, these mechanisms are still not fully understood. The characterization of the immunomodulatory roles of these antiviral proteins is essential to bridge the understanding between innate and adaptative immunity and is an expanding area of research that is essential to fully apprehend protective immune responses against IAV.

In this review, we also focused on the restriction factors present in other species of interest for IAV, namely pigs, bats and avian species. It is important to keep in mind that most of IAV strains circulating in humans arise from zoonotic introductions of avian-derived strains. As a consequence, after cross-species transmission and spill-over, avian-derived strains are not perfectly adapted to replicate in humans, where the viral polymerase activity is strongly impaired, missing crucial cofactors. There is also a lack of adaptation concerning the antagonism imposed by cellular restriction factors. Despite the poor genome annotation of poultry species, such as ducks, chickens and turkeys, or bats and pigs compared to that of humans, the vast majority of these proteins appear to be evolutionary conserved. Even if experimental data on these conserved proteins in all of these species are still lacking, recent studies pinpoint the conservation of the antiviral activity of some antiviral proteins such as MX1, IFITs or IFITM3. However, future studies are required to investigate whether each restriction factor described here are conserved in all these species of interest and whether their antiviral functions are similar to those of their mammalian counterparts.

With available treatments few and far between, focusing on understanding the molecular mechanisms of action of these known antiviral effectors is crucial to pave the way for the development of new therapeutic strategies against influenza. A particular focus should also be applied to the discovery of new antiviral inhibitors for the same reasons, taking advantage of state-of-the-art technologies, such as CRISPR/Cas9 screens, to lengthen the list of known host cell influenza inhibitors. Of note, results from large-scale siRNA and CRISPR screens tend to have little overlap, which is expected as screens can differ largely due to many variables such as cell types, viral strains and siRNA/sgRNA libraries used as well as multiple experimental variables which are harder to control. Therefore, it is of continued interest to perform these screens anew to discover as of yet unknown restriction factors.

The uncertainty as to when the next influenza pandemic will occur, with a vivid reminder being the current global situation due to the COVID-19 pandemic, should be initiative enough to push research forward in this field. We are not ready for the next influenza pandemic and the solution may well reside within our cells.
